# Changes in Ecosystem Services Value and Establishment of Watershed Ecological Compensation Standards

**DOI:** 10.3390/ijerph16162951

**Published:** 2019-08-16

**Authors:** Xin Gao, Juqin Shen, Weijun He, Fuhua Sun, Zhaofang Zhang, Xin Zhang, Chengcai Zhang, Yang Kong, Min An, Liang Yuan, Xiaocang Xu

**Affiliations:** 1 Business School, Hohai University, Nanjing 211100, China; 2 International Economic &Technical Cooperation and Exchange Center, Ministry of Water Resources, Beijing 100038, China; 3 College of Economics & Management, Three Gorges University, Yichang 443002, China; 4 Upper Yangtze river Economic Research Center/School of Economics, Chongqing Technology and Business University, Chongqing 400067, China

**Keywords:** watershed ecological compensation, ecosystem services value, land-use, South-to-North Water Transfer Project, Eastern Route, GIS and remote sensing technology

## Abstract

Ecological compensation standards and the allocation of compensation funds have always been the core issues of watershed ecological compensation. Due to the construction of the Eastern Route of the South-to-North Water Transfer Project (SNWTP), Jiangsu Province has paid a huge cost for the protection of water resources, and local economic development has been greatly affected. Therefore, this paper takes Jiangsu Province, the water source area of the Eastern Route of the SNWTP as an example, and combines a geographic information system (GIS) and remote sensing technology, using the ecosystem services value method to calculate the ecosystem services value of Jiangsu Province from 2005 to 2018. Then the change of this ecosystem services value in Jiangsu Province from 2015 to 2018 is taken as the basis for watershed ecological compensation standards of the Eastern Route. Through a compensation fund allocation model, watershed ecological compensation funds are allocated to four cities, Yangzhou, Huai’an, Suqian and Xuzhou, which are located along the Eastern Route of this SNWTP in Jiangsu Province. The results show that: (1) The ecosystem services value of Jiangsu Province has changed greatly. Urbanization and market environment of grain crops are the main reasons for this change; (2) the relationship between ecosystem services in Jiangsu Province is mainly synergistic; (3) Suqian receives US$24.73 million; Huai’an receives US$16.49 million; Yangzhou receives US$54.88 million and Xuzhou receives US$0.95 million in watershed ecological compensation, respectively. Watershed ecological compensation standards based upon the value of ecosystem services, and the allocation of compensation funds at the municipal level, are conducive to the improvement in efficiency of watershed ecological compensation in mainland China.

## 1. Introduction

As the source of life, the river basin has nurtured many brilliant civilizations, such as the ancient Egyptian civilization that flourished in the Nile River basin [1], the Babylonian civilization that bloomed along the Euphrates and Tigris rivers [2], the ancient Indian civilization that thrived besides the Indus and Ganges river [3], and the Chinese civilization that blossoms along the Yangtze and Yellow rivers [4]. It can be seen that the area which the basin flows through is often the birthplace of civilization and the center of the economy. In modern times, river basins also play the same role in cultural and economic development. Because of the geographical advantage and abundant ecological products and services in the basin [5,6], people prefer to migrate from other areas to this basin [7]. The population explosion in the basin has led to a large consumption of local ecological products and services, such as land, water, food, etc. [8,9]. This process of evolution is called urbanization [10]. The development of urbanization changes land-use patterns in the basin [11,12], which changes ecological processes, the ecological structure and ecological functions in the basin [13,14,15,16]. The promotion of urbanization stimulates the economic growth of the brain and the betterment of people’s lives [17,18]. However, with the acceleration of urbanization, the ecosystem in the basin has suffered tremendous damage, such as cross-regional water pollution [19,20,21,22], air pollution [23,24] etc. The frequent occurrence of environmental problems makes people re-examine the relationship between economic development and their ecological environment [25].

Due to the mobility of water resources, the use of water resources in the upstream area will affect the whole basin. If the upstream area carries on the rapid urbanization to promote economic development, it will be detrimental to water resources, which will affect the whole downstream area [26,27]. If the upstream area protects this water environment, it will affect local economic development. Then there is a contradiction between water resources conservation and economic development that needs to be solved urgently [28].

As a system, watershed ecological compensation has been widely used in mainland China, such as the Xin’an River [29], SNWTP [30], the Chishui River [31], etc. Its purpose is to adjust the contradiction between water protection and economic development, to promote cooperation between upstream and downstream areas in order to protect the watershed environment, and to achieve sustainable development [32]. According to mainland China’s environmental protection policy, upstream areas would protect water resources and provide good ecological products and services for downstream areas [33]. Protection of water resources in upstream areas will lead to the loss of enormous development opportunities. According to the basic principle of ecological compensation, “who benefits, who compensates” [34], the development opportunities lost in the upstream area could be compensated by the downstream area [35]. 

At present, watershed ecological compensation funds in mainland China come primarily from state revenue [36], and make it under great pressure. Now the Chinese government encourages the spontaneous implementation of watershed ecological compensation between upstream and downstream areas [30,37]. So, in order to promote the implementation of it, compensation standards and the allocation of funds have become core issues. 

So far, there are four main methods to determine compensation standards: (1) The contingent valuation method (CVM), which directly inquiries about the amount of acceptance (or payment) of various stakeholders in upstream and downstream areas for improving (or protecting) water resources by questionnaires [38]. It can be divided into two types: Willingness to pay (WTP) [39] and willingness to accept (WTA) [40]. It can easily obtain the information of residents’ willingness to accept and pay for resources, and can be used in a wide range, and can help avoid collecting and collating plenty of data. (2) The opportunity cost method, which regards the maximum economic benefit of the upstream area due to the loss of water resources protection as the final compensation standards [41,42]. It has the advantages of simple operation and wide application, especially when the socio-economic value of water resources cannot be directly estimated; this method can indirectly estimate its value. (3) Water resources value method. It means that upstream and downstream areas, as buyers and sellers in the market, making use of the relationship between supply and demand in the market to trade water resources [43,44]. 

Based on the law of supply and demand, it operates easily in practice, and can consider the interests of different regions of the basin. (4) The ecosystem services value method. This evaluates the value of various ecological services in the basin [45,46] to quantify the total value of ecological services in the upstream area, and then determines the final compensation standards [47,48]. The ecosystem service value method can accurately reflect the change of ecosystem service value. This is because the land area of different ecosystems can be obtained more accurately with the development of GIS and remote sensing technology. This can provide an objective reference for ecological compensation standards [49,50]. After determining the watershed ecological compensation standard, the allocation of watershed ecological compensation funds becomes the next core research issue. Previous studies mainly used water quantity or cross-section water quality as a reference index to determine the allocation scheme [51,52], and the research mainly focuses upon the allocation of compensation funds among different Provinces [31,53].

Although there are many methods to study watershed ecological compensation standards and allocation of compensation funds, there are some limitations: (1) CVM’s lack of objectivity will lead to large deviations in results. (2) The opportunity cost method and water resources value method require high accuracy and integrity of data, but these data are often difficult to obtain, which easily causes errors in the results. (3) The water resources value method needs a relatively stable and mature water resources market. However, considering China’s water market has not fully developed, this method will lead to an insufficient, unrealistic basis for the calculation results. After calculating the value of the ecosystem services, we need to allocate the ecological compensation funds reasonably. Although the current allocation of compensation funds has achieved some results, there are still the following limitations: (1) The allocation scheme based upon water quantity and cross-section water quality only cannot reflect other efforts made by upstream areas to protect water resources [51,52]. (2) The allocation scheme at large-scale provincial level [31,53] cannot reflect the Chinese government’s demand for the efficiency of watershed ecological compensation.

Therefore, in order to fix these research gaps, Jiangsu Province is taken as the research object, which is the water source of the Eastern Route of the SNWTP. The ecosystem services value method is used to analyze the change of ecosystem services value in Jiangsu Province, and takes it as a reference of final compensation standards. Combining with the fund allocation model at the municipal level, compensation funds are allocated to four cities located along the Eastern Route of the SNWTP (i.e., Yangzhou, Huai’an, Suqian and Xuzhou).

This paper mainly makes the following contributions: (1) With GIS and remote sensing technology, ecosystem services value in Jiangsu Province was estimated. (2) Spatial and temporal changes in ecosystem services value from 2005 to 2018 were analyzed. (3) Watershed ecological compensation standards of the Eastern Route were determined through changes in ecosystem service value. (4) Watershed ecological compensation funds of this Eastern Route were allocated at the municipal level.

The rest of this paper is structured as follows: Section 2 introduces the study area and data collection. Section 3 constructs the model of ecosystem services value, sensitivity, ecosystem services trade-off degree and the allocation model of the watershed ecological compensation funds. Section 4 shows the results of different models. Section 5 discusses the results and limitations. Section 6 arrives at conclusions and offers recommendations.

## 2. Study area and Data

### 2.1. Study Area

Jiangsu is one of the fastest growing Provinces in mainland China, which is located on the eastern coast of mainland China. Jiangsu Province ranks first in the comprehensive competitiveness of the regional economy, and is one of the most active Provinces in mainland China. From 2010 to 2018, the permanent urban population of Jiangsu Province has increased from 78.69 million to 80.29 million (+2%). GDP increased from US$6179.96 billion in 2010 to US$12814.29 billion in 2018.

**Figure 1:**
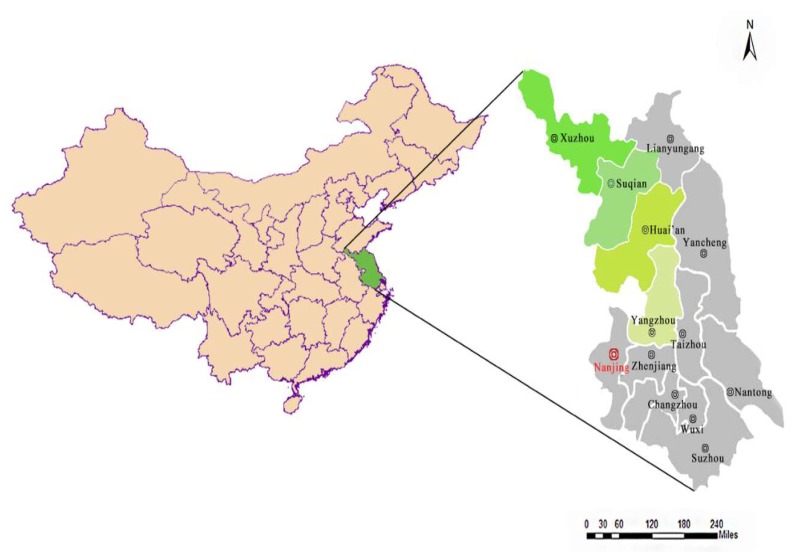
The map of the study area in mainland China.

Population explosion and economic development have caused great changes in land use patterns and ecosystem services. However, the construction of the Eastern Route of SNWTP has turned Jiangsu Province into a water source area. Therefore, in order to improve the water quality of this Eastern Route, Jiangsu Province has invested a large amount of funds to protect water resources. As a result, the economic development of Jiangsu Province has been greatly affected. In Jiangsu Province, cities along the East Route of the SNWTP (namely, Yangzhou, Huai’an, Suqian and Xuzhou) have made more efforts to maintain water quality. Meanwhile, the economic impact of them is more obvious than other cities. The study area is listed in Figure 1.

### 2.2. Data Collection and Basic Land-Use Classification

The land-use data (2005, 2010, 2015 and 2018) of Jiangsu Province in mainland China comes from the Resource and Environment Data Cloud Platform of the Chinese Academy of Sciences. These data are based on Landsat-8 remote sensing images, and are generated by manual visual interpretation. Data on the sown area and net profit per unit area of rice, wheat and maize in Jiangsu Province are derived from the China Statistical Yearbook and the Compilation of Cost-Benefit Data of National Agricultural Products. According to the classification criteria of the Chinese Academy of Sciences, land-use types include six primary types of cultivated land, forest land, grassland, water area, residential land and unused land, and 25 secondary types. 

## 3. Method and Models

### 3.1. Calculation of Per-Unit Ecosystem Services Value for Different Land-Use Types

#### 3.1.1. Calculation of Per-Unit Standard Value of Ecosystem Services

Xie et al. [54] believe the per-unit standard value of ecosystem services is the economic value of the grain crops produced by an average hectare of farmland in mainland China. The aim is to quantify the contribution of different ecosystems to ecosystem services. In this paper, the net profit of per-unit grain production of farmland ecosystem is regarded as per-unit standard ecosystem services value. The value of grain production in farmland is mainly obtained by accounting the economic value of three major grain products, which are rice, wheat and maize. The calculation formula is shown in Equation (1).

(1) $$D_{t}=A_{r,t}\times N_{r,t}+A_{w,t}\times N_{w,t}+A_{m,t}\times N_{m,t}$$

where $D_{t}$ represents the per-unit standard value of ecosystem services (US$·ha^−1^); $A_{r}$, $A_{w}$ and $A_{m}$ represent the percentage of the sown area of rice, wheat and maize in the total sown area; *t* refers to time; $N_{r,t}$, $N_{w,t}$ and $N_{m,t}$ refer to the net profit of rice, wheat and maize, respectively.

Because the sown area and the net profit of them are different from 2005 to 2018, we calculated four per-unit standard values of ecosystem services. The sown area and the net profit of different grain are shown in Table 1.

#### 3.1.2. Land-Use Classification

The calculation of ecosystem services is based upon the different land-use types, so proper land classification will help calculate ecosystem services value in the study area. As a land classification standard, Land-Use and Land-Cover Change (LUCC) is widely used in the world. Based on LUCC, the Chinese Academy of Sciences classified land in mainland China into six categories, which are arable land, woodland, grassland, water area, construction land and unused land. According to mainland China’s actual natural resources situation and research needs, Xie et al. [54] adjusted the criteria of LUCC classification and proposed a new land classification that has been widely used. The classification of Xie also divides the land into six categories and several subclasses, which are listed in Table 2. 

The remote sensing image data of Jiangsu Province from 2010 to 2018 obtained by the resource and environment data cloud platform actually follow LUCC. In order to convert it into Xie’s land classification, we reclassify the land-use types of Jiangsu Province in this paper. 

Because the difference is construction land and unused land in the first-level classification between two forms of classification, it is necessary to reclassify construction land and unused land. The unused land in Jiangsu Province is mainly saline-alkali land, swampland, bare land and bare rock texture. The ecosystem function of saline-alkali land is similar to the desert, so it is classified as desert. Similarly, marshes are classified as wetlands. Bare land, bare rock texture, urban and rural industrial and mining residential land are classified as bare land.

In the second-level classification, the differences are mainly reflected in forestland and grassland. Considering the climatic conditions in Jiangsu Province and the field survey of the Jiangsu Forestry Bureau, forestland and other woodlands in LUCC are classified as coniferous and broad-leaved, mixed forests; sparse woodland in LUCC is classified as shrub forest; grasslands with high, medium and low coverage are classified as shrubs and grasslands. Thus, the land-use classification of Jiangsu Province in this paper is listed in Table 3.

**Figure 2:**
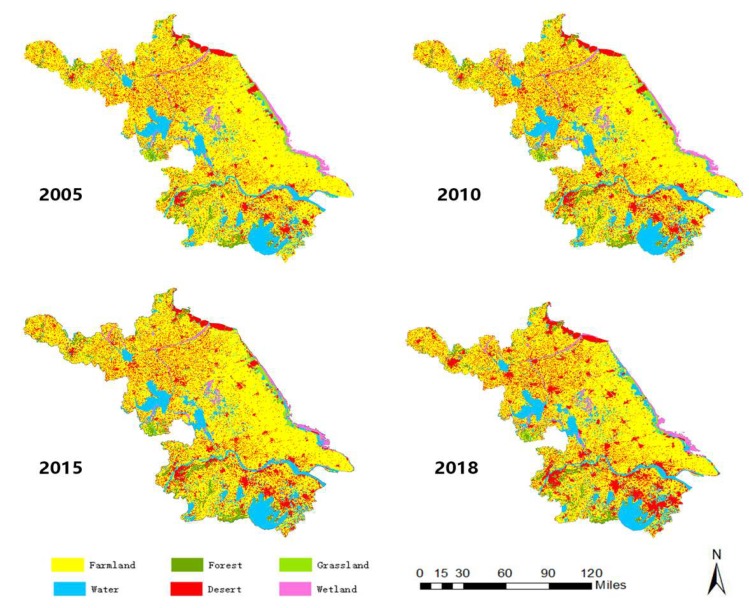
Spatial distribution of land-use in Jiangsu Province from 2005 to 2018.

The spatial-temporal distribution of land-use in Jiangsu Province in 2005, 2010, 2015 and 2018 are shown in Figure 2.

#### 3.1.3. The Locally-Adapted Ecosystem Services Value Coefficients in Jiangsu Province

The ecosystem services value coefficients ($B_{k,s}$) are adjusted according to land-use types in the study area, where *k* refers to the land-use type and *s* represents different ecosystem service. Xie et al. divide ecosystem services into four parts: Supply services, regulation services, support services and cultural services [54]. However, since the Eastern Route of the South-to-North Water Transfer Project (SNWTP) is in the first stage of construction, for security and strategic considerations, the Eastern Route Project is not open to the public at present. It is difficult to measure the cultural value of the Eastern Route Project by any appropriate methods. Therefore, in this paper, we will not study cultural services in the Eastern Route of the SNWTP. The locally-adapted ecosystem services value coefficients are shown in Table A1.

#### 3.1.4. Per-Unit Ecosystem Services Value of Jiangsu Province

Per-unit ecosystem services value is the basis of calculating the ecosystem services value. $VC_{s,k,t}$ is used to describe the per-unit ecosystem services value (US$ ha^−1^), where *s* represents the different ecosystem services, *k* represents the land-use type and *t* refers to time, t∈{2005, 2010, 2015, 2018}, and the calculation is shown in Equation (2).

(2) $$VC_{s,k,t}=D_{t}\times B_{k,s}$$

#### 3.1.5. Ecosystem Services Value of Jiangsu Province

According to Section 3.1.2, we classify the land of Jiangsu Province into six types and ten different ecosystem services (Table A1). We calculate each ecosystem service *s* in the set of ten ecosystem services *S* (Table A1) in Jiangsu Province for each land-use *k* in the set of six land-use types *K* (Table 3). In addition, the ecosystem services value ($ESV_{t}$) is calculated as the sum of the different ecosystem services in each land-use type in time *t*, and this ecosystem service value ($ESV_{t}$) in the currnet paper only includes the objective ecosystem service value of Jiangsu Province. The calculation of $ESV_{t}$ is shown in Equations (3) and (4).

(3) $$ESV_{s,\;k,\;t}=\alpha_{k,t}\times VC_{s,k,t}$$

(4) $$ESV_{t}=\underset{k}{\sum }\underset{s}{\sum }ESV_{s,\;k,\;t}{}_{\;}$$

where $\alpha_{k,t}$ refers to the area (in hectares) of different land-use *k* at time *t* in the study area.

### 3.2. Sensitivity Analysis

In order to measure the dependence of ecosystem services on per-unit ecosystem services value, we use the coefficient of sensitivity (*CS*) to measure it [55,56,57,58]. The *CS* is usually applied based on the change of per-unit ecosystem services value, for example, a ±50% change in the per-unit ecosystem services value for service *s* and land-use type *k*, at each time *t*. The calculation formula is Equation (5):

(5) $$CS_{s,k,t}=\left|\frac{\left(ES{V^{\prime }}_{s,k,t}-ESV_{s,\;k,\;t}\right)/ESV_{\;t}}{(VC\prime_{s,k,t}-VC_{s,k,t})/VC_{s,k,t}}\right|$$

where $ESV_{s,\;k,\;t}$ and $ES{V^{\prime }}_{s,k,t}$ refer to the ecosystem services value before and after the change of the per-unit ecosystem services value, respectively. $VC_{s,k,t}$ and ${VC\prime }_{s,k,t}$ represent these per-unit ecosystem services values before and after the ±50% change. In this study, we replace the percentage change of the per-unit ecosystem services value with *x*. It means that a +50% change of per-unit value equals to x=1.5, while −50% of change equals to x=0.5.

According to the research of Aschonitis et al. [59], if the initial $VC\prime_{s,k,t}$ changing with the *x* and VC of the remaining land-use is constant, then the values’ change of $VC\prime_{s,k,t}$ and $ES{V^{\prime }}_{s,k,t}$ in Equation (5) will equal to them in Equations (6) and (7):

(6) $$VC\prime_{s,k,t}=x\times VC_{s,k,t}$$

(7) $$ES{V^{\prime }}_{s,k,t}=ESV_{s,k,t}-\left(1-x\right)\times VC_{s,k,t}\times \alpha_{k,t}$$

Taking Equations (6) and (7) into Equation (5), Equation (5) can be adjusted in accordance with the following:

(8) $$CS_{s,k,t}=\left|\frac{\left(ES{V^{\prime }}_{s,k,t}-ESV_{s,\;k,\;t}\right)/ESV_{\;t}}{(VC\prime_{s,k,t}-VC_{s,k,t})/VC_{s,k,t}}\right|=\left|\frac{VC_{s,k,t}\times \alpha_{k,t}}{ESV_{\;t}}\right|$$

If $CS_{s,k,t}>1$, it represents that *ESV* is resilient to *VC*; if $CS_{s,k,t}<1$, it represents that *ESV* is inelastic to *VC*. The $CS_{s,k,t}$ value obtained from Equation (8) means the accuracy of *VC*. The greater the absolute value of $CS_{s,k,t}$, the higher its accuracy. 

### 3.3. Ecosystem Services Trade-off Degree

Ecosystem Services Trade-off Degree (ESTD) is a method based on linear fitting of data to reflect the direction and degree of interaction among ecosystem services [60,61]. The aim of ESTD is to evaluate the interaction of ecosystem services change as a whole in the study area. ESTD can be calculated with Equation (9):

(9) $$ESTD_{ij}=\frac{ESC_{ib}-ESC_{ia}}{ESC_{jb}-ESC_{ja}}$$

In Equation (9), $ESTD_{ij}$ represents the ecosystem services trade-off degree between ecosystem services *i* and *j*, $ESC_{ib}$ the value of ecosystem services with *i* at time *b*, $ESC_{ia}$ the value of ecosystem services with *i* at the time *a,* $ESC_{jb}$ and $ESC_{ja}$ the same. ESTD represents the balance and direction of the interaction between the two ecosystem services [62]. When ESTD is negative, the relationship between them is a trade-off; when ESTD is positive, the relationship between them is synergy [63]. The absolute value of ESTD represents the degree of change in ecosystem services of type *i* compared with that of type *j*.

### 3.4. Watershed Ecological Compensation Standards

#### 3.4.1. Watershed Ecological Compensation Scope

The ecosystem services value is the highest value of watershed ecological compensation, that is, the upper limit of watershed ecological compensation. Based upon the analysis of 10 types of ecosystem services in the Eastern Route of SNWTP, ecosystem functions serving the region and the whole country are included in the compensation scope. Considering that the value of supply services in ecosystem services needs to be converted into monetary value in the market, and the spillover scope of functions such as gas regulation and biodiversity maintenance is global, the division of compensation responsibility cannot be realized, so it cannot be included in the final compensation. Therefore, watershed ecological compensation measures five services function, including climate regulation, hydrological regulation, environmental purification, hydrological regulation, soil conservation and nutrient cycling maintenance, which are included in the compensation scope. 

#### 3.4.2. Ecological Compensation Priority Sequence

The priority of ecological compensation (ECPS) refers to the ratio of the non-market value of ecosystem services per unit area to GDP per unit area in a region, which indicates the urgency of obtaining ecological compensation in different regions [64]. $ECPS_{i}$ can be calculated with Equations (10) and (11):

(10) $$ECPS_{i}=\frac{V_{i}}{G_{i}}$$

(11) $$V_{i}=V_{total}*\left(S_{i}/S_{total}\right)$$

where $ECPS_{i}$ represents the ecological compensation priority sequence in city *i*; $V_{total}$ and $V_{i}$ represent a non-market value of ecosystem services in Jiangsu Province and a non-market value of ecosystem services in the city *i*, respectively; $S_{i}$ and $S_{total}$ represent the area of city *i* and the total area of Jiangsu Province, respectively; $G_{i}$ refers to GDP per unit area. The greater the ECPS, the stronger the impact of ecological compensation on regional economic development. Therefore, ecological compensation should be given priority.

#### 3.4.3. Calculation of Watershed Ecological Compensation Standards

In order to alleviate the urgent development needs of regions with lower GDP per unit area and reduce the risk of a rapid reduction of regional ecological value, priority should be given to areas with a high demand for watershed ecological compensation. Based on this, the regional differences of watershed ecological compensation standards are reflected by the demand intensity coefficient and ecological value conversion coefficient. The demand intensity coefficient is expressed by the normalized result of watershed ecological compensation priority, and the conversion coefficient of ecological value is 15% [65]. The specific calculation is shown in Equations (12) and (13).

(12) $$R_{i}=V_{i}\times h\times m_{i}$$

(13) $$m_{i}=2\times \tan^{-1}\frac{ECPS_{i}}{\pi }$$

where $R_{i}$ represents the total amount of regional watershed ecological compensation, *h* the conversion coefficient of ecological value, $m_{i}$ the demand intensity coefficient in city *i*.

## 4. Results

### 4.1. Changes in Land-Use

**Figure 3:**
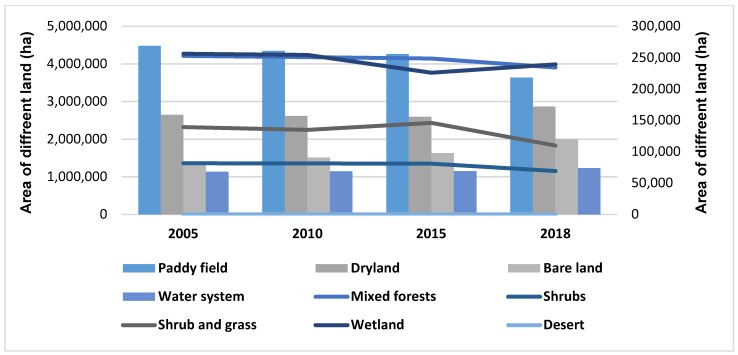
The changes of land-use in Jiangsu Province.

From 2005 to 2018, great changes in land-use took place in Jiangsu Province. The bare land increased by 48.10%, and the dryland and water area increased by 8.22% and 8.56%, respectively. Desert land has changed from 0 hectares to 100 hectares, which is the emergence of saline-alkali land in Jiangsu Province in 2018. However, other types of land area decreased, including grassland, which decreased by 21.27%, the largest decline; paddies decreased by 18.7%; shrub forests decreased by 15.16%; coniferous-broad-leaved mixed forests and wetlands decreased by 7.23% and 6.54%, respectively. The changes in land-use are shown in Figure 3. 

According to land-use classification in this paper, bare land contains three parts: Bare land, bare rock texture, urban and rural industrial and mining residential land. From 2005 to 2018, bare land increased the most from 1.35 million hectares to 1.99 million hectares. Among them, the largest change was urban, industrial and mining residential land, from 1.35 million hectares in 2005 to 1.99 million hectares in 2018, an increase of 47.8%. The large increase in it shows that the urbanization in Jiangsu Province has accelerated from 2005 to 2018. At the same time, due to the large increase in urban, industrial and mining residential land, the area of land of other types will be reduced, such as farmland, grassland, forest and water system.

### 4.2. Changes in the Value of Ecosystem Services

#### 4.2.1. Changes in the Value of Ecosystem Services in Jiangsu Province from 2005 to 2018

**Figure 4:**
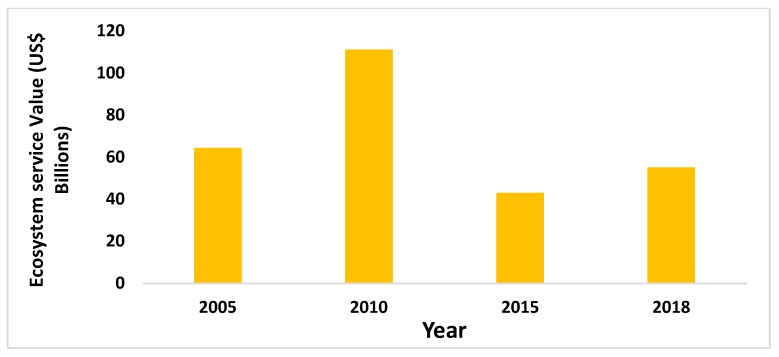
The Value of Ecosystem Services from 2005 to 2018.

Figure 4 shows the value of ecosystem services in Jiangsu Province from 2005 to 2018. Among them, the value of ecosystem services in 2010 was the highest, reaching US$11.11 billion; in 2015, the value of ecosystem services was the lowest, only US$4.398 billion (−61.32%). From 2005 to 2018, the value of ecosystem services in Jiangsu Province represented a process of rapid growth first, then a swift decline, and finally a slight increase. Overall, the value of the ecosystem services in Jiangsu Province reached US$6.44 billion in 2005 and US$5.51 billion in 2018 (−14.38%). 

**Figure 5:**
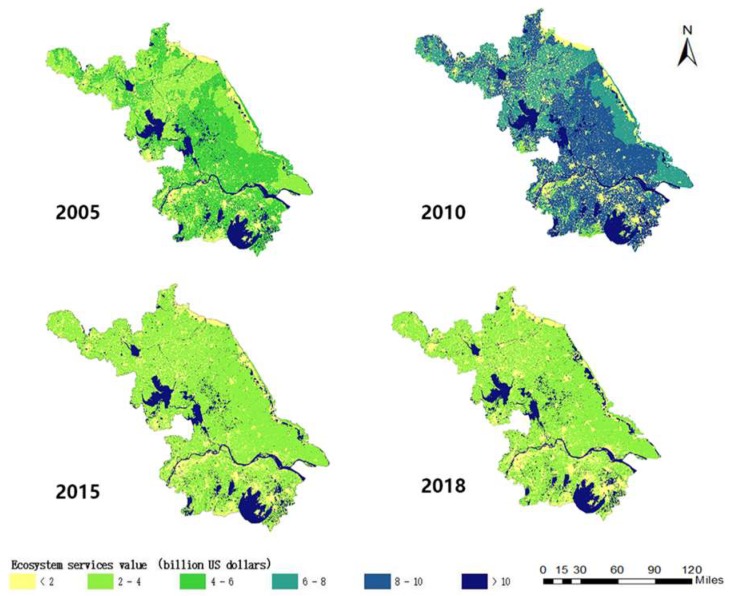
Spatial distribution of the ecosystem services value from 2005 to 2018.

Spatial distribution of ecosystem services value in four years is shown in Figure 5. From Figure 5, we can find that the high-value areas of the ecosystem services are mainly distributed in the central and southern parts of Jiangsu Province, as well as in the river and lake regions. The ecosystem services value in 2010 is the highest. The value in 2005 is higher than that in 2015 and 2018. 

#### 4.2.2. Changes in the Value of Different Ecosystem Services

**Figure 6:**
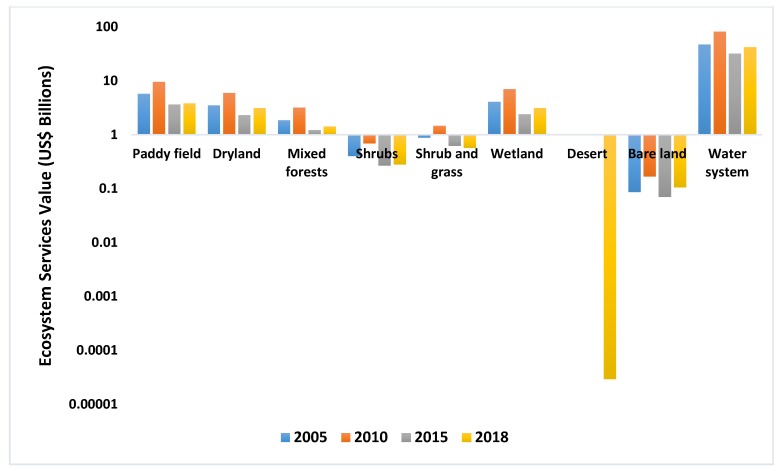
The Value of Different Ecosystem Services.

The value of different ecosystem services is shown in Figure 6. It is clear that the most valuable ecosystem services are water system, paddy, wetland, dryland and mixed forests. Among them, the water system contains the most value, and reached a maximum of US$82.76 billion in 2010, then began to decline, reaching a minimum of US$32.34 billion in 2015. From 2005 to 2018, the water system as the most valuable ecosystem service has decreased (−10.76%). Paddies (from US$5.78 billion to US$3.86 billion, −33.24%), wetlands (from US$4.12 billion to US$3.16 billion, −23.17%), drylands (from US$3.55 billion to US$3.16 billion, −11.04%) and mixed forests (from US$1.88 billion to US$1.43 billion, −23.74%). Other ecosystem services, such as desert, shrubs, shrub and grass and bare land, can only provide a very limited value of ecosystem services. Desert provides the least value, only US$29,340, but the desert that appeared in Jiangsu Province in 2018 was saline-alkali soil. From 2005 to 2018, the ecosystem services value of shrubs and grass decreased from US$0.89 billion to US$0.57 billion (−35.28%), and shrubs dropped from US$0.4 billion to US$0.28 billion (−30.26%), respectively. However, bare land’s ecosystem services value increased from US$0.086 billion to US$0.11 billion (+21.75%), which was the only increase in all ecosystem services. 

#### 4.2.3. Changes in Individual Ecosystem Services Value of Different Land Uses

**Figure 7:**
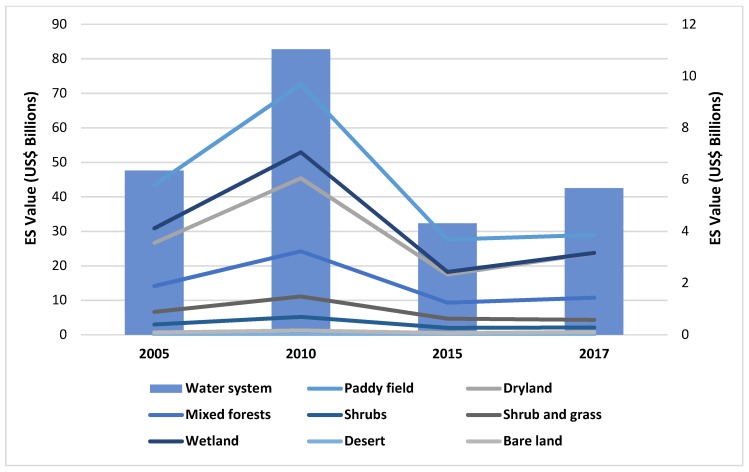
Individual Ecosystem Services Value of Different Land-uses.

According to Section 4.1, we can find that the land-use types in Jiangsu Province were changed greatly from 2005 to 2018. Among them, the area of bare land, water, desert and dryland increased, while the area of shrub and grass, paddies, shrubs, mixed forests and wetland, decreased. Thus, the changes in land use influenced the individual ecosystem services, which are shown in Figure 7.

Bare land increased most in these land-use types. The area of bare land in 2005 was 1.35 million hectares, but it has increased to 1.99 million hectares (+48.10%). The rapid increase in bare land area will lead to a sharp reduction of other land-use areas, such as farmland, forest, grassland and wetland. According to the classification in this paper, farmland contains two types: Paddies and dryland. The area of the paddies dropped a lot, but that of the dryland increased slightly. Overall, the area of farmland dropped 0.62 million hectares (−8.76%). Since the area of farmland changed in these years, its value dropped US$2.31 billion. Meanwhile, forests include mixed forests and shrubs. From 2005 to 2018, the area of mixed forest and shrub decreased by 7.23% and 15.16%, respectively. Overall, the forest area decreased by 30,642 hectares (−0.92%). Consequently, the value of forest ecosystem services decreased by about US$0.57 billion. The most noticeable decrease in the area of grassland occurred from 139,416 hectares in 2005 to 109,758 hectares in 2018 (−21.27%). The value of grassland has also decreased by US$0.31 billion, due to the sharp decrease in the grassland area. By comparison, the area of bare land, water system and desert increased correspondingly. The water system is the major contributor of ecosystem services in all land-use types in Jiangsu Province. The area of the water system was 113,235 hectares, and it contributed US$47.67 billion to Jiangsu Province in 2005. In the following years, the area of Jiangsu’s water system did not fluctuate dramatically. However, its value increased slightly by 2010, and then dropped sharply to the lowest point in 2015, finally rising slowly to the present state. The main reason for this trend is the change in per-unit ecosystem services value. The desert appeared mainly as saline-alkali land in 2018, and provided only US$29,340. 

### 4.3. Sensitivity Analysis

According to Equation (8), we can get sensitivity coefficients in 2005, 2010, 2015 and 2018, which are listed in Table A2, Table A3, Table A4 and Table A5. For all types of land use and different ecosystem services in this paper, the absolute value of all sensitivity coefficients was close to zero. It means that the value of different ecosystem services in Jiangsu Province is stable to the changes of per-unit ecosystem services in different land-use types. Among them, only the sensitivity coefficient of water supply is negatively provided by paddy, and it means water supply’s ecosystem services value is inelastic to per-unit ecosystem services of paddy; other sensitivity coefficients of different ecosystem services are positive. It shows that the value of other ecosystem services is elastic to per-unit ecosystem services in different land-use types. The sensitivity coefficient of hydrologic adjustment provided by the water system is the highest in all coefficients, where for a single percentage point change in per-unit ecosystem services, the ecosystem services value changed about 60%.

### 4.4. Analysis of Ecosystem Services Trade-off Degree

The correlation analysis of ecosystem services is to analyze the relationship between ecosystem services from the whole-time span. In order to further study the degree and direction of interaction between ecosystem services in different time periods, we introduced the ESTD model to quantify and evaluate the relationship between ecosystem services in Jiangsu Province.

**Figure A1:**
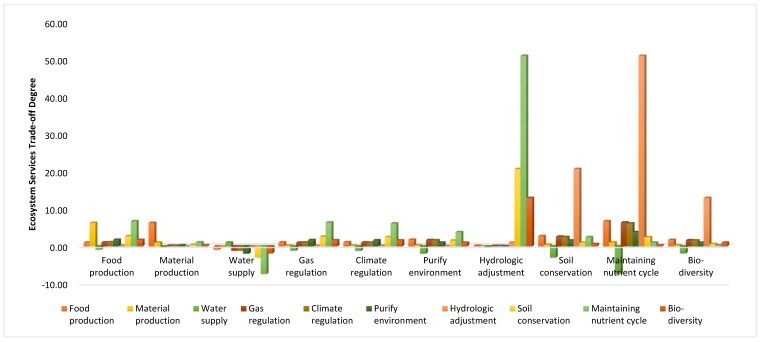
Ecosystem Services Trade-off Degree in 2005–2018.

From 2005 to 2018, ESTD is listed in Table A6 and Figure A1. According to Table A6, there are 100 groups of synergies among ecosystem services, of which 18 groups are negative, 82 groups are positive, and 82% are synergistic. This shows that synergy is the dominant relationship among ecosystem services in Jiangsu Province. Among them, synergy mainly exists in all ecological services, except water supply. The synergistic degree between maintaining the nutrient cycle and hydrological regulation is the highest, and the trade-off degree between maintaining the nutrient cycle and water resources supply is also the highest.

At 12 December 2014, the Eastern Route of the SNWTP began to operate. Water resources originally belonging to Jiangsu Province transferred to the lower reaches of the Shandong Province. This brings about changes in the value of ecosystem services in Jiangsu Province. 

**Figure A2:**
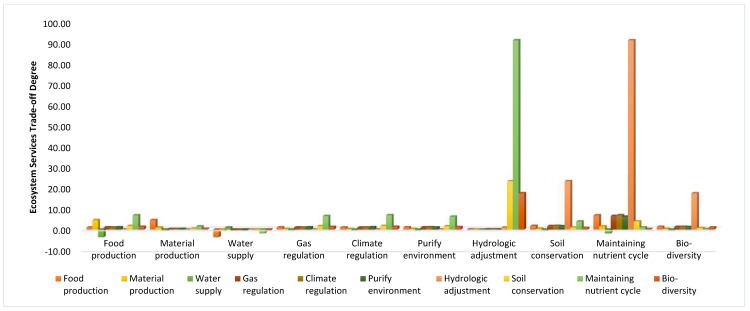
Ecosystem Services Trade-off Degree in 2005–2015.

**Figure A3:**
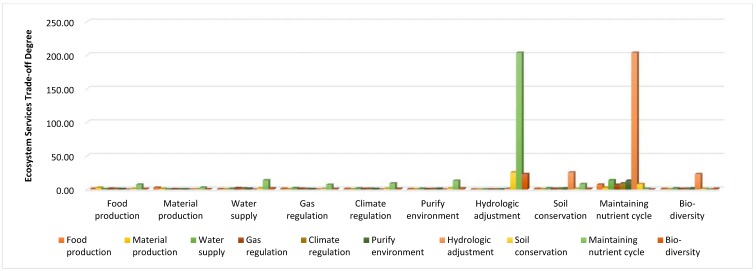
Ecosystem Services Trade-off Degree in 2015–2018.

In order to reflect the changes in ecosystem services in Jiangsu Province, ESTDs from 2005 to 2015 and from 2015 to 2018 were calculated. ESTDs in Jiangsu Province from 2005 to 2015 are shown in Table A7 and Figure A2, while ESTDs from 2015 to 2018 are shown in Table A8 and Figure A3. Through Figure A2 and Figure A3, it can be found that both the degree and direction of ecosystem services interaction in Jiangsu Province have changed after the operation of the Eastern Route of SNWTP.

From 2005 to 2015, the proportion of trade-offs and synergies between ecosystem services in Jiangsu Province was consistent with that in 2005–2018. There were 18 trade-offs and 82 synergies. All trade-offs exist in the water supply of supply services, while the other parts are synergistic. Among them, the synergy between the maintaining nutrient cycle and hydrological conditions is the highest, but it is higher than that in 2005–2018. In 2005–2015, the highest trade-off was between water supply and food production, not water supply and nutrient maintenance in 2005–2018.

From 2015 to 2018, the proportion of trade-offs and synergies between ecosystem services in Jiangsu Province changed greatly, compared with that in 2005–2018. From 2015 to 2018, all ecosystem services in Jiangsu Province were synergistic. Among them, the highest synergy is to maintain the nutrient cycle and hydrological regulation, while the lowest synergy is hydrological regulation and raw material production.

According to the degree of interaction of ecosystem services in Jiangsu Province, the absolute value of ESTD increases, while the changes in synergy degree of different types of ecosystem services are different. In regulation services, the synergy between hydrological regulation and other ecosystem services increases; in contrast, in supply services, the trade-off relationship between water resources supply and other ecosystem services has changed into synergy. In addition, the synergy between food production and other ecosystem services is reduced in supply services; the same happens in gas and climate regulation services. The synergy between soil and water conservation and food and raw material production decreased, and the synergy with gas and climate regulation also decreased.

### 4.5. Watershed Ecological Compensation Fund Allocation Scheme in Different Cities

Since the Eastern Route of the SNWTP began to operate at the end of 2014, a large number of watershed ecological protection works have been carried out in cities along the Eastern Route of the SNWTP in Jiangsu Province, including Yangzhou, Huai’an, Suqian and Xuzhou. In order to transfer good water to Shandong Province, these cities have paid a huge cost for watershed ecological protection. Therefore, watershed ecological compensation for the Eastern Route of the SNWTP should also be carried out for these four cities. Since the environmental protection is concentrated after 2015, watershed ecological compensation standards for the Eastern Route are aimed at the period from 2015 to 2018.

According to Section 3.4, five ecosystem services are included in the final compensation scope, namely climate regulation, hydrological regulation, environmental purification, soil conservation and maintaining the nutrient cycle. Thus, from 2015 to 2018, the non-market value of Jiangsu Province is listed in Table A9. It is clear that this non-market value was US$37.012 billion in 2015 and US$47.383 billion in 2018, having increased by US$10.371 billion. From 2015 to 2018, theoretically, the total amount of watershed ecological compensation of the Eastern Route was US$10.371 billion. However, this amount of compensation, which cannot reach the final compensation standards, is only the upper limit of watershed ecological compensation standards.

From Equations (10) to (13), we can get the watershed ecological compensation priority sequence and watershed ecological compensation scheme in different cities along the Eastern Route. The results are listed in Table A10, Table A11 and Table A12. The priority sequence and the demand intensity coefficients are consistent, whereas funds allocation is different from them. Finally, Suqian receives US$24.73 million; Huai’an receives US$16.49 million; Xuzhou receives US$10.95 million and Yangzhou receives US$8.41 million.

## 5. Discussions

Watershed ecological compensation has become an important system to promote the coordinated development of regional economy and ecological environment. Then, the watershed ecological compensation standard and the fund allocation scheme have become the core content to ensure the implementation of this system.

### 5.1. Major Drivers of Ecosystem Services Value

The change of land-use type is one of the main reasons for the change in the value of ecosystem services in Jiangsu Province. Through Section 4.1, we can find that from 2005 to 2018, bare land (+48.1%), water system (+8.56%) and dry land (+8.22%) increased significantly in Jiangsu Province. The most obvious increase in bare land was contributed by urban, industrial and mining residential land. The dramatic increase in urban residential land reflects the rapid urbanization and industrialization in Jiangsu Province, which, as one of mainland China’s major economic Provinces, perennially ranks in the top three of the wealthiest Provinces. According to the China Statistical Yearbook of 2018, Jiangsu’s GDP increased from 1.88 trillion yuan in 2005 to 8.59 trillion yuan in 2018, following Guangdong Province and ranking second in the country. The rapid economic development in Jiangsu Province makes it attractive, not only to enterprises, but also to job seekers. According to the Jiangsu Statistical Yearbook of 2018, the total population in Jiangsu Province increased from 75.88 million in 2005 to 82.93 million in 2018. At the same time, the urbanization rate of Jiangsu Province reached 69.61% in 2018, 10.03 percentage points higher than the national average (59.58%) and ranked fifth in mainland China. Through the above data, we can find that the rapid urbanization and industrialization in Jiangsu Province has proven to be a catalyst for its booming economy. Because of the rapid economic development, there are more employment opportunities in Jiangsu Province, which attracts a large number of migrant workers. The increase in population will lead to a rapid increase in urban and residential land. Under the constraints of unchanged total land area, in order to increase urban and residential land, the governments of Jiangsu Province have to convert other forms of land into urban and residential land, which will lead to drastic changes in land-use types in Jiangsu. From 2005 to 2018, grassland area in Jiangsu Province decreased by 21.27%, while paddies, wetlands and forest areas also decreased to a large extent. According to the reclassification of land-use types in this paper, urban and residential land is classified as bare land. Compared with other types of land, bare land can provide the lowest value of ecosystem services. Therefore, with a large number of land types, such as paddies, wetlands, forests and grasslands transformed into bare land, the value of ecosystem services in Jiangsu Province has declined significantly.

Per-unit standard ecosystem services value is another important factor affecting the value of ecosystem services in Jiangsu Province. The value of ecosystem services is obtained by multiplying the per-unit ecosystem services value and land area. The per-unit ecosystem services value is calculated on the basis of the market value of three major grain crops (rice, maize and wheat). In different years, since the price of the three major grain crops is subject to the economic environment, the value of ecosystem services is indirectly affected by it. According to Section 4.2.1, we can find that the ecosystem services value of Jiangsu Province reached the highest value in 2010, based on the reason that the net profit of the three major grain crops was the highest. From 2005 to 2018, the net profit per unit of rice, wheat and maize has changed to some extent. Net profit per unit of rice is relatively balanced, but net profit per unit of wheat and maize varies greatly. In 2005 and 2010, net profit per hectare of wheat was $284.38 and $375.97, but in 2015 and 2018, this net profit per hectare was only US$2.88 and US$7.44. In contrast, the net profit per unit of maize changed more. US$11.55, US$716.27, US$−172.97 and US$−476.30 (Table 1) were the net profits per hectare of maize in the four years of 2005, 2010, 2015 and 2018, respectively. It can be found that the change of net profit will directly affect the per-unit standard ecosystem services value. Therefore, the final per-unit standard ecosystem services value in four years were US$340.03, US$586.53, US$227.52 and US$279.52, respectively. The main reasons for such great changes in the price of grain crops are as follows: (1) The income of migrant workers is higher than that of growing grain. Rural households may own only 0.2 hectares of arable land per household, so the maximum net profit of grain crops is US$17.082.

According to the Jiangsu Statistical Yearbook, the average wage of the employees engaged in agriculture, forestry, animal husbandry and fishery in 2010 was US$5,886.92 per year. As the income of migrant workers is obviously higher than that of growing grain, a large number of farmers choose to work in cities; (2) the cost of grain increases year by year; after all, seed cost, fertilizer cost, agricultural machinery use cost and labor cost are increasing year by year, which makes the unit net profit reduce; (3) the market price of grain is low, and in recent years, with the impact of imported grain, domestic grain prices have been depressed. Based on the above reasons, the price of food crops has changed dramatically, and the per-unit standard ecosystem services value has also changed constantly.

The purpose of accounting ecosystem services value is to monetize ecosystem services, and then calculate the economic value of ecosystem services, so that we can understand the evolution of the ecosystem and formulate a sustainable development mode that can consider both the economy and the environment. However, the above analysis shows that the accounting of ecosystem services value is not only affected by land-use area, but also by the net profit of food crops. The relationship between the ecosystem and economic system is complex, and reflects a state of interaction.

### 5.2. Trade-offs and Synergies of Ecosystem Services in the Eastern Route of SNWTP

In this paper, the ESTD model is used to study the relationship between ecosystem services in Jiangsu Province. The three periods are 2005–2018, 2005–2015 and 2015–2018. The overall trend of ESTD in 2005–2018 is similar to that in 2005–2015. Among the 100 results, 82 groups are synergies and 18 groups are trade-offs. Synergies are the dominant relationship among ecosystem services in Jiangsu Province. These 18 trade-offs are all caused by the supply of water resources. In addition, comparing the results of ESTD in 2005–2015 and 2015–2018, we can find that the relationship between ecosystem services are synergistic. Human factors have become the main cause of ecosystem services change in Jiangsu Province. On 12 December 2014, the Eastern Route Project of SNWTP came into operation, and a large number of water resources in Jiangsu Province began to be transferred to Shandong Province, which indicates an improvement in the water resources supply and service capacity of Jiangsu Province. At the same time, due to the construction of the Eastern Route Project, many reservoirs, water conveyance channels and other water conservancy facilities emerged in Jiangsu Province, greatly increasing the area of water. Due to the water resources transferred from Jiangsu Province to Shandong Province, the available water resources in Jiangsu Province are reduced, which weakens the role of ecosystem services in other parts, such as gas regulation, postponement regulation, environmental purification, hydrological regulation, etc. In addition, the river channel in some areas of the Jiangsu Province has been cut down, the diversion conditions have been changed and the groundwater level has been lowered, which has also brought adverse effects on the irrigation diversion in some areas. Meanwhile, the Eastern Route Project has changed the temporal and spatial characteristics of surface water and groundwater, and has an impact on the biochemical properties of the soil in Jiangsu Province. It leads to the reduction of food production capacity in supply services. At the same time, this Eastern Route Project cuts off the connectivity of the local ecosystem, interrupts the relationship between different species, and then has a negative impact on the biodiversity of Jiangsu Province.

### 5.3. The Relationship between Ecosystem Services Value and Watershed Ecological Compensation Standards

Watershed ecological compensation, as an important economic incentive policy, has been implemented in most countries. It requires the monetization of ecosystem products and services to facilitate transactions. Ecosystem services value just meets the needs of watershed ecological compensation. These quantitative economic values will become an important reference and basis for determining watershed ecological compensation standards.

According to previous scholars’ research on watershed ecological compensation standards, when they use the ecosystem services value method to determine watershed ecological compensation standards, they basically regard the ecosystem services value in one year in the research area as the final watershed ecological compensation standards [32], which value has some mistakes. 

Firstly, according to Tacconi’s definition of ecological compensation, “ecological compensation is a transparent system for conditional payment of resource providers for environmental gain services”. Through the definition, it is clear that we should compensate for the improvement in the environmental effect. If ecosystem services value is regarded by scholars as the final compensation standard, they cannot reflect the condition of ecological compensation. Therefore, this paper argues that watershed ecological compensation standards should be the change of ecosystem services value in different years, rather than the value of ecosystem services in one year. Secondly, if the ecosystem services value is taken as the final compensation standard, it will lead to huge amounts of compensation fund beyond what people can afford. This is unfavorable to the implementation of the watershed ecological compensation system.

Therefore, the ecosystem services value in one year cannot be directly used as the final watershed ecological compensation standards. At the same time, the change of ecosystem services value in different years can reflect Jiangsu Province’s efforts in protecting the water environment. Taking the change of ecosystem services value as the basis of determining the watershed ecological compensation standards, it is a method that conforms to the definition of watershed ecological compensation.

### 5.4. Watershed Ecological Compensation Standards and Fund Allocation

The watershed ecological compensation standard is the basis of fund allocation. The core of fund allocation needs to be given attention on both the intra-generation equity and efficiency. In watershed ecological compensation, intra-generation equity is mainly embodied in the following two parts: Firstly, the equity between the compensating subject and the compensated subject; secondly, the equity of the compensation fund distribution. For the first part, intra-generational equity is mainly reflected in the equity of the right to development. Since the operation of the Eastern Route of the SNWTP, Jiangsu Province takes various measures to protect water resources, such as restricting the development of polluting enterprises, adjusting industrial structure, and establishing ecological protection zones. This will inevitably affect the further development of Jiangsu’s economy. If Shandong Province (compensating subject), does not make watershed ecological compensation for Jiangsu Province (compensated subject), then the development right of Jiangsu Province has been violated. Therefore, the implementation of the watershed ecological compensation system of the Eastern Route Project protects the right to development of Jiangsu Province, and reflects the intergenerational equity between the two provinces. For the second part, based on the current situation of watershed ecological compensation in China, we consider the compensation fund allocation in the Eastern Route of the SNWTP, which reflects the intra-generation equity and efficiency. However, the current allocation of watershed ecological compensation funds mainly carries on the equal allocation in the provincial scope. This seemingly absolutely fair distribution method neglects the investment of various provinces and municipalities in ecological protection. It has led to a realistic problem in the compensation fund allocation in China: Regions with an obvious increase in the ecosystem services value receive little or no compensation, while regions with less increase in the ecosystem services value receive more compensation funds. Therefore, according to the previous principle of equal distribution, the fund allocation among Provinces reflects the lack of intra-generational equity and low compensation efficiency. 

In order to solve the current problems in the compensation fund allocation, we allocate compensation funds based on the increment of ecosystem service value, which is a distribution method with efficiency as the main factor and equity as the supplement. This method overcomes the inequity and inefficiency caused by the equal allocation. Therefore, this method can better combine intra-generation equity and efficiency to achieve better watershed ecological compensation.

In the Eastern Route, since not all of the cities in Jiangsu Province contribute to the improvement of water quality, Shandong Province only needs to compensate the contributing cities, including Yangzhou, Suqian, Huai’an and Xuzhou. According to Section 4.5, Yangzhou, as the water source of the Eastern Route Project, has received the lowest compensation, which is different from common sense. After consulting the statistical yearbook of Yangzhou, we found that watershed protection of Yangzhou started before the operation of the Eastern Route and yielded positive results. Thus, from 2015 to 2018, Yangzhou’s ecosystem services stabilized at a better level, without significant improvements being made. As ecological compensation is mainly for the growth of ecosystem service value, Yangzhou finally receives the least. On the contrary, Suqian, Huai’an and Xuzhou have embarked multiple environmental protection projects since the Eastern Route Project came into operation, such as tailrace diversion projects. Since the projects contributed to the improvement of ecosystem services in these cities, the final allocation of watershed ecological compensation accounts for a larger portion of it.

An appropriate funds allocation scheme of watershed ecological compensation can not only reflect the equity and efficiency among different regions, but also guarantee the long-term implementation of the watershed ecological compensation system. In previous research on compensation funds allocation in mainland China [31,53], scholars mainly focused on the allocation at the provincial level. However, as to how to allocate compensation funds to cities on the basis of equity and efficiency, there is no effective method for implementation at present. Watershed ecological compensation has entered an in-depth development stage in mainland China. It is no longer limited to large-scale compensation, but to small-scale compensation. The purpose is to ensure the balanced development between the upstream and downstream regions, and to reflect its equity and efficiency The differentiated allocation of compensation funds at the urban level also reflects precise compensation.

### 5.5. Limitations

This paper has the following limitations: (1) It only considers the supply value of ecosystem services, but does not consider its demand value. According to economic theory, the transaction price will be determined by supply and demand. This is an interesting direction for future research. (2) The self-consumption of ecosystem services value is not considered. In determining watershed ecological compensation standards between Jiangsu and Shandong Province, the compensation amount should deduct self-consumption of ecosystem services in Jiangsu Province. (3) The spillover effect of ecosystem services value among different cities is not considered when allocating watershed ecological compensation funds. In future research, the spillover effect of ecological services can be dealt with by constructing the ecological value matrix. (4) The long-term and sustainability of ecological compensation cannot be ignored in the study of ecological compensation, and intra-generation equity and efficiency are important parts of it. How to guarantee intra-generation equity and improve the efficiency of watershed ecological compensation will be the direction of future research.

## 6. Conclusions

The construction of the Eastern Route of SNWTP has turned Jiangsu Province into a water conservation area. In order to determine watershed ecological compensation standards and the fund allocation scheme of the Eastern Route, we first calculated the changes of land use and ecosystem services value from 2005 to 2018 in Jiangsu Province using a per-unit standard value of the ecosystem services method; secondly, we determined the reliability of the calculation results of this ecosystem services value in Jiangsu Province by sensitivity analysis; thirdly, the interaction of ecosystem services in Jiangsu Province was described by ESTD; finally, the allocation model of watershed ecological compensation funds was constructed to determine the final allocation scheme. The main conclusions are as follows: 

(1) From 2005 to 2018, urbanization is an important reason for the great changes in land-use types in Jiangsu Province.

(2) From 2005 to 2018, the ecosystem services value in Jiangsu Province changed greatly, and reached its maximum in 2010. The land-use type and net profit of grain crops are the main driving factors that restrict the ecosystem services value in Jiangsu Province.

(3) Synergy is the main relationship between ecosystem services in Jiangsu Province.

(4) Taking the change of ecosystem service value as the basis to determine watershed ecological compensation can better reflect the environmental gain effect in the definition of ecological compensation.

(5) According to the compensation fund allocation scheme in this paper, Suqian receives US$24.73 million; Huai’an receives US$16.49 million; Xuzhou receives US$10.95 million and Yangzhou receives US$8.41 million.

Based on the analysis in this paper, the following recommendations are made to improve watershed ecological compensation in the Eastern Route of the SNWTP:

(1) According to the change of ecosystem service value, the government needs to design a more scientific urbanization development plan. 

(2) Establishing a complete spatial monitoring system is helpful to improve the accuracy of the ecosystem services value.

(3) The government needs to increase scientific and technological investment in crop production, increase the output value of major grain crops, reduce their costs and then increase their net profits.

(4) The government needs to take the value of ecosystem services as the basis of accounting ecological compensation standards, and allocate ecological compensation funds at the municipal level, which will help to improve the efficiency of watershed ecological compensation.

## Figures and Tables

**Table 1:** The sown area and the net profit of rice, wheat and maize.

	Rice		Wheat		Maize	
Year	Sown Area (ha)	Net Profit (US$/ha)	Sown Area (ha)	Net Profit (US$/ha)	Sown Area (ha)	Net Profit (US$/ha)
**2005**	2,209,330	3938.7	7,641,200	1906.35	370,240	77.4
**2010**	2,234,160	8588.625	7,619,580	2520.3	403,700	4801.5
**2015**	2,291,590	6229.5	7,745,040	289.8	451,680	−1159.5
**2018**	2,275,960	6680.925	7,601,250	748.05	470,490	−3192.9

Note: These data were collected from the Compilation of Cost-Benefit Data of National Agricultural Products. In these files, the net profit of different grain crops is equal to the total output value of the product, minus the total cost. Among them, the gross output value includes the output value of the main products and the output value of by-products. Total cost includes production cost, labor cost and land cost. The net profit of grain crops in different years is based on fixed-base data.

**Table 2:** The land-use classification of Xie.

First Classification	Secondary Classification
**Farmland**	Dryland; Paddy
**Forest**	Coniferous forest; broad-leaved forest; Coniferous-broad-leaved mixed forest; Shrub
**Grassland**	Grassland; Shrub and grass; Meadow
**Wetland**	Wetland
**Desert**	Desert; Bare land.
**Water**	River system; Glacier.

**Table 3:** Land-use classification of Jiangsu Province.

First Classification	Secondary Classification	Specification
**Farmland**	Dryland	
	Paddy	
**Forest**	Mixed coniferous and broad-leaved forests	Forest land; Other woodlands
	Shrubs	Sparse woodland; Shrubs
**Grassland**	Shrub and grass	Grasslands with different coverage
**Wetland**	Wetland	Marshes; Shoals
**Desert**	Desert	Saline-alkali land
	Bare land	Bare land; bare rock texture; urban and rural industrial and mining residential land
**Water**	Water system	Rivers; Lakes; Reservoir; Swag

## Appendix A

**Table A1:** The ecosystem services value coefficients for Jiangsu Province.

Ecosystem Classification		Supply Service			Regulatory Services				Support Services		
First Classification	Second Classification	Food Production	Material Production	Water Supply	Gas Regulation	Climate Regulation	Purify Environment	Hydrologic Adjustment	Soil Conservation	Maintaining Nutrient Cycle	Bio- Diversity
**Farmland**	①	1.36	0.09	−2.63	1.11	0.57	0.17	2.72	0.01	0.19	0.21
	②	0.85	0.4	0.02	0.67	0.36	0.1	0.27	1.03	0.12	0.13
**Forest**	③	0.31	0.71	0.37	2.35	7.03	1.99	3.51	2.86	0.22	2.6
	④	0.19	0.43	0.22	1.41	4.23	1.28	3.35	1.72	0.13	1.57
**Grassland**	⑤	0.38	0.56	0.31	1.97	5.21	1.72	3.82	2.4	0.18	2.18
**Wetland**	⑥	0.51	0.5	2.59	1.9	3.6	3.6	24.23	2.31	0.18	7.87
**Desert**	⑦	0.01	0.03	0.02	0.11	0.1	0.31	0.21	0.13	0.01	0.12
	⑧	0	0	0	0.02	0	0.1	0.03	0.02	0	0.02
**Water**	⑨	0.8	0.23	8.29	0.77	2.29	5.55	102.24	0.93	0.07	2.55

Note: ① Paddy; ② Dryland; ③ Mixed coniferous and broad-leaved forests; ④ Shrubs; ⑤ Shrub and grass; ⑥ Wetland; ⑦ Desert; ⑧ Bare land; ⑨ Water.

**Table A2:** The sensitivity coefficients for ecosystem services values in the Jiangsu Province in 2005.

Classification		Supply Service		Regulatory Services				Support Services				Average
First Classification	Second Classification	Food Production	Material Production	Water Supply	Gas Regulation	Climate Regulation	Purify Environment	Hydrologic Adjustment	Soil Conservation	Maintaining Nutrient Cycle	Bio- Diversity	
**Farmland**	①	0.0322	0.0021	−0.0622	0.0263	0.0135	0.0040	0.0643	0.0002	0.0045	0.0050	0.0090
	②	0.0119	0.0056	0.0003	0.0094	0.0050	0.0014	0.0038	0.0144	0.0017	0.0018	0.0055
**Forest**	③	0.0004	0.0009	0.0005	0.0031	0.0094	0.0027	0.0047	0.0038	0.0003	0.0035	0.0029
	④	0.0001	0.0002	0.0001	0.0006	0.0018	0.0006	0.0014	0.0007	0.0001	0.0007	0.0006
**Grassland**	⑤	0.0003	0.0004	0.0002	0.0015	0.0038	0.0013	0.0028	0.0018	0.0001	0.0016	0.0014
**Wetland**	⑥	0.0007	0.0007	0.0035	0.0026	0.0049	0.0049	0.0328	0.0031	0.0002	0.0107	0.0064
**Desert**	⑦	0.0000	0.0000	0.0000	0.0000	0.0000	0.0000	0.0000	0.0000	0.0000	0.0000	0.0000
	⑧	0.0000	0.0000	0.0000	0.0001	0.0000	0.0007	0.0002	0.0001	0.0000	0.0001	0.0001
**Water**	⑨	0.0048	0.0014	0.0496	0.0046	0.0137	0.0332	0.6117	0.0056	0.0004	0.0153	0.0740
**Average**		0.0056	0.0013	−0009	0.0053	0.0058	0.0054	0.0802	0.0033	0.0008	0.0043	0.0111

Note: ① Paddy; ② Dryland; ③ Mixed coniferous and broad-leaved forests; ④ Shrubs; ⑤ Shrub and grass; ⑥ Wetland; ⑦ Desert; ⑧ Bare land; ⑨ Water.

**Table A3:** The sensitivity coefficients for ecosystem services values in the Jiangsu Province in 2010.

Classification		Supply Service		Regulatory Services				Support Services				Average
First Classification	Second Classification	Food Production	Material Production	Water Supply	Gas Regulation	Climate Regulation	Purify Environment	Hydrologic Adjustment	Soil Conservation	Maintaining Nutrient Cycle	Bio- Diversity	
**Farmland**	①	0.0312	0.0021	−0.0603	0.0254	0.0131	0.0039	0.0624	0.0002	0.0044	0.0048	0.0087
	②	0.0117	0.0055	0.0003	0.0092	0.0050	0.0014	0.0037	0.0142	0.0017	0.0018	0.0054
**Forest**	③	0.0004	0.0009	0.0005	0.0031	0.0093	0.0026	0.0046	0.0038	0.0003	0.0034	0.0029
	④	0.0001	0.0002	0.0001	0.0006	0.0018	0.0005	0.0014	0.0007	0.0001	0.0007	0.0006
**Grassland**	⑤	0.0003	0.0004	0.0002	0.0014	0.0037	0.0012	0.0027	0.0017	0.0001	0.0016	0.0013
**Wetland**	⑥	0.0007	0.0007	0.0035	0.0026	0.0048	0.0048	0.0325	0.0031	0.0002	0.0106	0.0063
**Desert**	⑦	0.0000	0.0000	0.0000	0.0000	0.0000	0.0000	0.0000	0.0000	0.0000	0.0000	0.0000
	⑧	0.0000	0.0000	0.0000	0.0002	0.0000	0.0008	0.0002	0.0002	0.0000	0.0002	0.0002
**Water**	⑨	0.0048	0.0014	0.0499	0.0046	0.0138	0.0334	0.6155	0.0056	0.0004	0.0154	0.0745
**Average**		0.0055	0.0012	−0.0006	0.0052	0.0057	0.0054	0.0803	0.0033	0.0008	0.0043	0.0111

Note: ① Paddy; ② Dryland; ③ Mixed coniferous and broad-leaved forests; ④ Shrubs; ⑤ Shrub and grass; ⑥ Wetland; ⑦ Desert; ⑧ Bare land; ⑨ Water.

**Table A4:** The sensitivity coefficients for ecosystem services values in the Jiangsu Province in 2015.

Classification		Supply Service		Regulatory Services				Support Services				Average
First Classification	Second Classification	Food Production	Material Production	Water Supply	Gas Regulation	Climate Regulation	Purify Environment	Hydrologic Adjustment	Soil Conservation	Maintaining Nutrient cycle	Bio- Diversity	
**Farmland**	①	0.0307	0.0020	−0.0593	0.0250	0.0129	0.0038	0.0613	0.0002	0.0043	0.0047	0.0086
	②	0.0117	0.0055	0.0003	0.0092	0.0049	0.0014	0.0037	0.0141	0.0016	0.0018	0.0054
**Forest**	③	0.0004	0.0009	0.0005	0.0031	0.0092	0.0026	0.0046	0.0038	0.0003	0.0034	0.0029
	④	0.0001	0.0002	0.0001	0.0006	0.0018	0.0005	0.0014	0.0007	0.0001	0.0007	0.0006
**Grassland**	⑤	0.0003	0.0004	0.0002	0.0015	0.0040	0.0013	0.0030	0.0019	0.0001	0.0017	0.0014
**Wetland**	⑥	0.0006	0.0006	0.0031	0.0023	0.0043	0.0043	0.0290	0.0028	0.0002	0.0094	0.0057
**Desert**	⑦	0.0000	0.0000	0.0000	0.0000	0.0000	0.0000	0.0000	0.0000	0.0000	0.0000	0.0000
	⑧	0.0000	0.0000	0.0000	0.0002	0.0000	0.0009	0.0003	0.0002	0.0000	0.0002	0.0002
**Water**	⑨	0.0049	0.0014	0.0504	0.0047	0.0139	0.0337	0.6217	0.0057	0.0004	0.0155	0.0752
**Average**		0.0054	0.0012	−0.0005	0.0052	0.0057	0.0054	0.0806	0.0033	0.0008	0.0042	0.0111

Note: ① Paddy; ② Dryland; ③ Mixed coniferous and broad-leaved forests; ④ Shrubs; ⑤ Shrub and grass; ⑥ Wetland; ⑦ Desert; ⑧ Bare land; ⑨ Water.

**Table A5:** The sensitivity coefficients for ecosystem services values in the Jiangsu Province in 2018.

Classification		Supply Service		Regulatory Services				Support Services				Average
First Classification	Second Classification	Food Production	Material Production	Water Supply	Gas Regulation	Climate Regulation	Purify Environment	Hydrologic Adjustment	Soil Conservation	Maintaining Nutrient cycle	Bio- Diversity	
**Farmland**	①	0.0251	0.0017	-0.0485	0.0205	0.0105	0.0031	0.0502	0.0002	0.0035	0.0039	0.0070
	②	0.0123	0.0058	0.0003	0.0097	0.0052	0.0015	0.0039	0.0149	0.0017	0.0019	0.0057
**Forest**	③	0.0004	0.0008	0.0004	0.0028	0.0084	0.0024	0.0042	0.0034	0.0003	0.0031	0.0026
	④	0.0001	0.0002	0.0001	0.0005	0.0015	0.0004	0.0012	0.0006	0.0000	0.0006	0.0005
**Grassland**	⑤	0.0002	0.0003	0.0002	0.0011	0.0029	0.0010	0.0021	0.0013	0.0001	0.0012	0.0010
**Wetland**	⑥	0.0006	0.0006	0.0031	0.0023	0.0044	0.0044	0.0294	0.0028	0.0002	0.0096	0.0057
**Desert**	⑦	0.0000	0.0000	0.0000	0.0000	0.0000	0.0000	0.0000	0.0000	0.0000	0.0000	0.0000
	⑧	0.0000	0.0000	0.0000	0.0002	0.0000	0.0010	0.0003	0.0002	0.0000	0.0002	0.0002
**Water**	⑨	0.0050	0.0014	0.0517	0.0048	0.0143	0.0346	0.6377	0.0058	0.0004	0.0159	0.0772
**Average**		0.0049	0.0012	0.0008	0.0047	0.0052	0.0054	0.0810	0.0033	0.0007	0.0040	0.0111

Note: ① Paddy; ② Dryland; ③ Mixed coniferous and broad-leaved forests; ④ Shrubs; ⑤ Shrub and grass; ⑥ Wetland; ⑦ Desert; ⑧ Bare land; ⑨ Water.

**Table A6:** Ecosystem Services Trade-off Degree in Jiangsu Province from 2005 to 2018.

Contents	Supply Service		Regulatory Services				Support Services			
	Food Production	Material Production	Water Supply	Gas Regulation	Climate Regulation	Purify Environment	Hydrologic Adjustment	Soil Conservation	Maintaining Nutrient Cycle	Bio- Diversity
**Food production**	1.0000	6.2749	−0.9038	1.0521	1.0958	1.7717	0.1320	2.7370	6.7404	1.7143
**Material production**	6.2749	1.0000	−0.1440	0.1677	0.1746	0.2823	0.0210	0.4362	1.0742	0.2732
**Water supply**	−0.9038	−0.1440	1.0000	−1.1641	−1.2124	−1.9603	−0.1461	−3.0284	−7.4580	−1.8968
**Gas regulation**	1.0521	0.1677	−1.1641	1.0000	1.0415	1.6840	0.1255	2.6015	6.4067	1.6294
**Climate regulation**	1.0958	0.1746	−1.2124	1.0415	1.0000	1.6169	0.1205	2.4978	6.1514	1.5645
**Purify environment**	1.7717	0.2823	−1.9603	1.6840	1.6169	1.0000	0.0745	1.5449	3.8045	0.9676
**Hydrologic adjustment**	0.1320	0.0210	−0.1461	0.1255	0.1205	0.0745	1.0000	20.7276	51.0457	12.9824
**Soil conservation**	2.7370	0.4362	−3.0284	2.6015	2.4978	1.5449	20.7276	1.0000	2.4627	0.6263
**Maintaining nutrient cycle**	6.7404	1.0742	−7.4580	6.4067	6.1514	3.8045	51.0457	2.4627	1.0000	0.2543
**Bio-diversity**	1.7143	0.2732	−1.8968	1.6294	1.5645	0.9676	12.9824	0.6263	0.2543	1.0000

**Table A7:** Ecosystem Services Trade-off Degree in Jiangsu Province from 2005 to 2015.

Contents	Supply Service		Regulatory Services				Support Services			
	Food Production	Material Production	Water Supply	Gas Regulation	Climate Regulation	Purify Environment	Hydrologic Adjustment	Soil Conservation	Maintaining Nutrient Cycle	Bio- Diversity
**Food production**	1.0000	4.5469	−3.6747	1.0477	0.9912	1.0999	0.0751	1.7467	6.8561	1.3097
**Material production**	4.5469	1.0000	−0.8082	0.2304	0.2180	0.2419	0.0165	0.3842	1.5078	0.2880
**Water supply**	−3.6747	−0.8082	1.0000	−0.2851	−0.2697	−0.2993	−0.0204	−0.4753	−1.8658	−0.3564
**Gas regulation**	1.0477	0.2304	−0.2851	1.0000	0.9461	1.0498	0.0716	1.6672	6.5440	1.2501
**Climate regulation**	0.9912	0.2180	−0.2697	0.9461	1.0000	1.1097	0.0757	1.7623	6.9171	1.3213
**Purify environment**	1.0999	0.2419	−0.2993	1.0498	1.1097	1.0000	0.0682	1.5881	6.2335	1.1907
**Hydrologic adjustment**	0.0751	0.0165	−0.0204	0.0716	0.0757	0.0682	1.0000	23.2695	91.3346	17.4472
**Soil conservation**	1.7467	0.3842	−0.4753	1.6672	1.7623	1.5881	23.2695	1.0000	3.9251	0.7498
**Maintaining nutrient cycle**	6.8561	1.5078	−1.8658	6.5440	6.9171	6.2335	91.3346	3.9251	1.0000	0.1910
**Bio-diversity**	1.3097	0.2880	−0.3564	1.2501	1.3213	1.1907	17.4472	0.7498	0.1910	1.0000

**Table A8:** Ecosystem Services Trade-off Degree in Jiangsu Province from 2015 to 2018.

Contents	Supply Service		Regulatory Services				Support Services			
	Food Production	Material Production	Water Supply	Gas Regulation	Climate Regulation	Purify Environment	Hydrologic Adjustment	Soil Conservation	Maintaining Nutrient Cycle	Bio- Diversity
**Food production**	1.0000	2.6427	0.5233	1.0363	0.7931	0.5521	0.0353	0.8973	7.1783	0.8097
**Material production**	2.6427	1.0000	0.1980	0.3921	0.3001	0.2089	0.0133	0.3395	2.7162	0.3064
**Water supply**	0.5233	0.1980	1.0000	1.9803	1.5155	1.0550	0.0674	1.7146	13.7172	1.5472
**Gas regulation**	1.0363	0.3921	1.9803	1.0000	0.7653	0.5327	0.0340	0.8658	6.9267	0.7813
**Climate regulation**	0.7931	0.3001	1.5155	0.7653	1.0000	0.6961	0.0445	1.1314	9.0510	1.0209
**Purify environment**	0.5521	0.2089	1.0550	0.5327	0.6961	1.0000	0.0639	1.6252	13.0018	1.4665
**Hydrologic adjustment**	0.0353	0.0133	0.0674	0.0340	0.0445	0.0639	1.0000	25.4499	203.6004	22.9648
**Soil conservation**	0.8973	0.3395	1.7146	0.8658	1.1314	1.6252	25.4499	1.0000	8.0001	0.9024
**Maintaining nutrient cycle**	7.1783	2.7162	13.7172	6.9267	9.0510	13.0018	203.6004	8.0001	1.0000	0.1128
**Bio-diversity**	0.8097	0.3064	1.5472	0.7813	1.0209	1.4665	22.9648	0.9024	0.1128	1.0000

**Table A9:** The Ecosystem Services (Non-market Value) in Jiangsu Province from 2015 to 2018.

Year	Climate Regulation	Purify Environment	Hydrologic Adjustment	Soil Conservation	Maintaining Nutrient Cycle	Total
2015	2.197	2.090	31.163	1.260	0.303	37.012
2018	2.597	2.664	40.161	1.613	0.348	47.383

Note: Billion dollars.

**Table A10:** Ecological priority sequence in different cities in Jiangsu Province.

City	GDP Per Unit Area (Million Dollars/Hectare)	Non-Market Value Per Unit Area (Million Dollars/Hectare)	Priority Sequence	Rank
**Yangzhou**	0.1139	0.0156	0.1373	3
**Huai’an**	0.0580	0.0121	0.2088	2
**Suqian**	0.0387	0.0103	0.2663	1
**Xuzhou**	0.0875	0.0092	0.1052	4

**Table A11:** Ecological priority sequence and demand intensity coefficient in different cities in Jiangsu Province.

	Priority Sequence	Rank	Demand Intensity Coefficient	Rank
**Yangzhou**	0.1373	3	0.0874	3
**Huai’an**	0.2088	2	0.1328	2
**Suqian**	0.2663	1	0.1692	1
**Xuzhou**	0.1052	4	0.0670	4

**Table A12:** Actual ecological compensation in different cities in Jiangsu Province.

	Area (ha)	Total Area (ha)	Percentage of Area	Ecological Compensation	Rank
				(Million Dollars)	
**Yangzhou**	663,400	10,720,000	6.19%	8.41	4
**Huai’an**	855,500		7.98%	16.49	2
**Suqian**	1,007,200		9.40%	24.73	1
**Xuzhou**	1,125,800		10.50%	10.95	3
